# Correction: LINC01410-miR-532-NCF2- NF-kB feedback loop promotes gastric cancer angiogenesis and metastasis

**DOI:** 10.1038/s41388-021-01796-4

**Published:** 2021-06-28

**Authors:** J. X. Zhang, Z. H. Chen, D. L. Chen, X. P. Tian, C. Y. Wang, Z. W. Zhou, Y. Gao, Y. Xu, C. Chen, Z. S. Zheng, H. W. Weng, S. Ye, D. Xie, S. Peng

**Affiliations:** 1 State Key Laboratory of Oncology in South China; Collaborative Innovation Center for Cancer Medicine, Sun Yat-Sen University Cancer Center, Guangzhou, China; 2 Department of Oncology & Department of Gastroenterology and Hepatology, the First Affiliated Hospital, Sun Yat-Sen University, Guangzhou, China; 3 Department of Urology, the First Affiliated Hospital, Sun Yat-Sen University, Guangzhou, China; 4 Zhongshan School of Medicine, Sun Yat-Sen University, Guangzhou, China; 5 Department of Nasopharyngeal Cancer, Sun Yat-Sen University Cancer Center, Guangzhou, China

Correction to: *Oncogene*

10.1038/s41388-018-0162-y

Following the publication of the above article, the authors noted several errors: (1) Fig. 1: The images of wound-healing assay in Fig. 1a were captured in low magnification. The authors replace these images in a higher magnification; The image of transwell assay for the “miR-532-5p group” of SGC-7901 cell line in Fig. 1b was misused and has been replaced. (2) Fig. 2: The image of transwell assay for AGS cell line in Fig. 2c was misused and has been replaced. (3) Fig. 3: the WB result of p54 protein of MNK-45 cell (right) was mistakenly displayed as a duplicate of SGC-7901 cell (left) in Fig. 3j and has been replaced. (4) Fig. 5: the images of transwell assay for “sh-NC group” and “sh-LINC01410#1 group” of MNK-45 cell line in Fig. 5a, “sh-LINC01410#2 group” of SGC-7901 cell line in Fig. 5b, “sh-NC group” of MNK-45 cell line in Fig. 5d, were misused and have been replaced; the image of HUVECs tube-formation assay for “LINC01410 group” of AGS cell line in Fig. 5c was accidentally misused and has been replaced. The authors confirm that the errors do not affect the results or conclusions of the study, and apologize for any inconvenience caused. The corrected figures are provided below.

Also, please note that the author ‘Ming Kuang (6)’ has been removed from the author list.

**Fig. 1:**
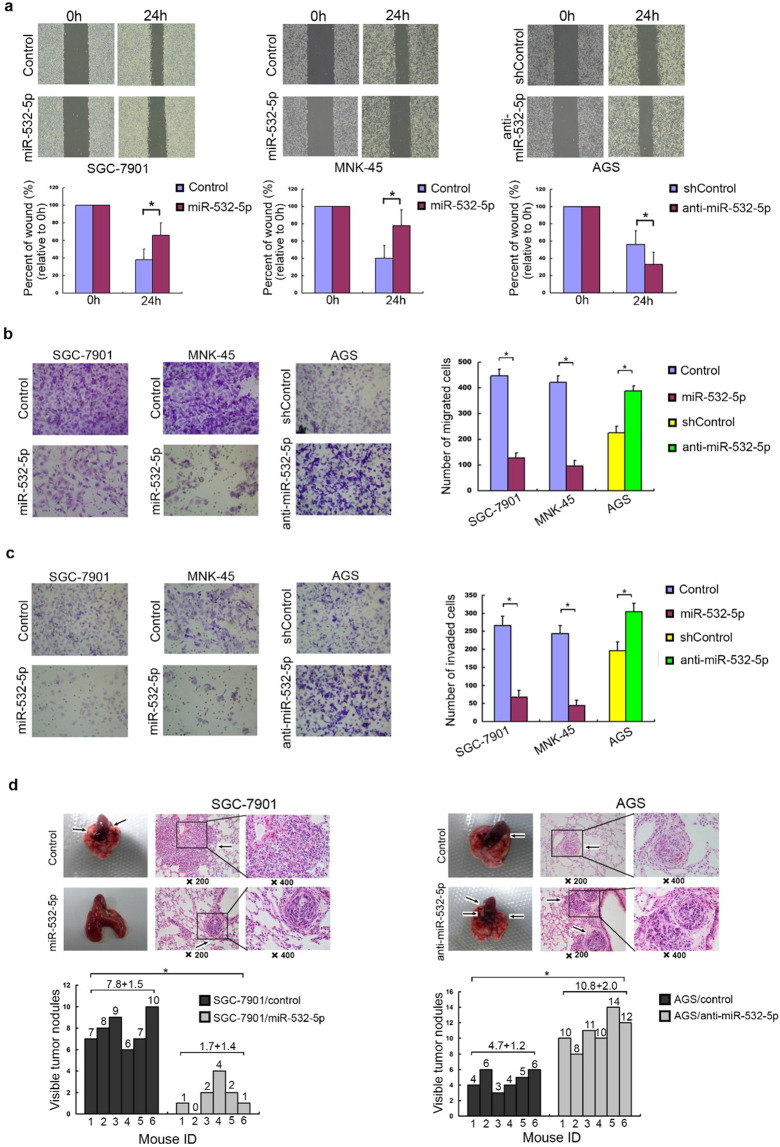
miR-532-5p inhibits gastric cancer metastasis in vitro and in vivo. **a** The wound healing rate in miR-532-5p-transfected SGC7901 and MNK-45 cells was largely inhibited, while enhanced in miR532-5p-silenced AGS cells. **b** The number of migrated cell was significantly decreased in miR-532-5p-overexpressing SGC-7901 and MNK-45, while increased in miR-532-5p-silenced AGS cells, as determined by transwell migration assay. **c** The number of invaded cell was decreased in miR-532-5p-overexpressing SGC-7901 and MNK45, while increased in miR-532-5p-silenced AGS cells, as assessed by Matrigel invasion assay. **d** miR-532-5p inhibits tumor metastasis in vivo. Upper panel: (Left) Representative bright-field imaging of the lungs; (Right) hematoxylin and eosin (H&E) staining was performed on serial sections of metastatic tumors and normal lung. Arrows: lesions of lung. Lower panel: the number of nodules was qualified on lungs of SCID mice (*n* = 6 per group) 6 weeks after tail vein injection of SGC-7901/miR-532-5p or SGC-7901/control, and AGC/anti-miR532-5p or AGC/shcontrol cells.

**Fig. 2:**
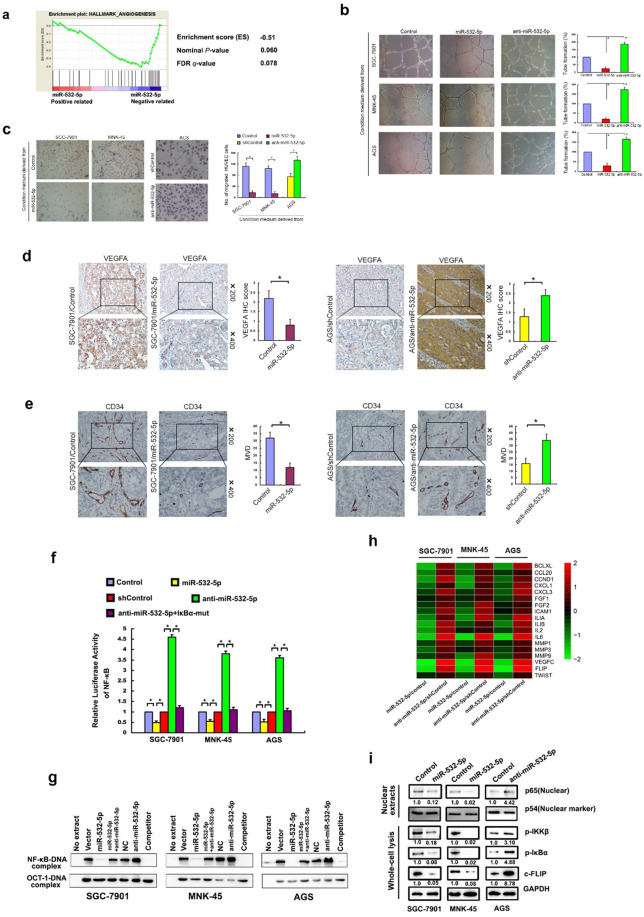
The inhibitory effects of miR-532-5p on tumor angiogenesis and NF-κB activity of GC cells. **a** GSEA plot showed miR-532-5p level was inversely correlated with angiogenesis gene signatures in the TCGA the stomach adenocarcinoma (STAD) data set. The abilities of in vitro capillary tube formation (**b**) and migration (**c**) of HUVEC were significantly decreased after incubating with culture medium of miR-532-5p-transfected GC cell, while potentially strengthened after incubating with culture medium of miR-532-5psilenced GC cell. The VEGFA levels (**d**) and MVD (**e**) in tumor tissue of nude mice models with subcutaneous implantation of GC were noticeably reduced when miR-532-5p was upregulated in SGC-7901 cells, while largely increased when miR-532-5p was downregulated in AGS cells. **f** Luciferase-reported NF-κB activity was decreased in miR-532-5p-overexpressing, while increased in miR-5325p-silenced GC cells. **g** EMSA indicated NF-κB activity dramatically decreased in miR-532-5p-transduced cells but increased in miR-5325p-inhibited cells. OCT-1 DNA-binding complexes served as a control. **h** qRT-PCR analysis showed an apparent overlap between miR532-5p-regulated gene expression and NF-κB-dependent gene expression. The pseudocolors represent the intensity scale of miR-5325p versus control or anti-miR-532-5p versus shcontrol vector, which was generated by a log 2 transformation. **i** Western blotting assay showed that nuclear NF-κB/p65, p-IKK-β, p-IκBα, and c-FLP expressions were decreased in miR-532-5p-overexpressing SGC7901 and MNK-45, while potentially increased in miR-532-5psilenced AGS cells. p54 was used as a nuclear loading control.

**Fig. 3:**
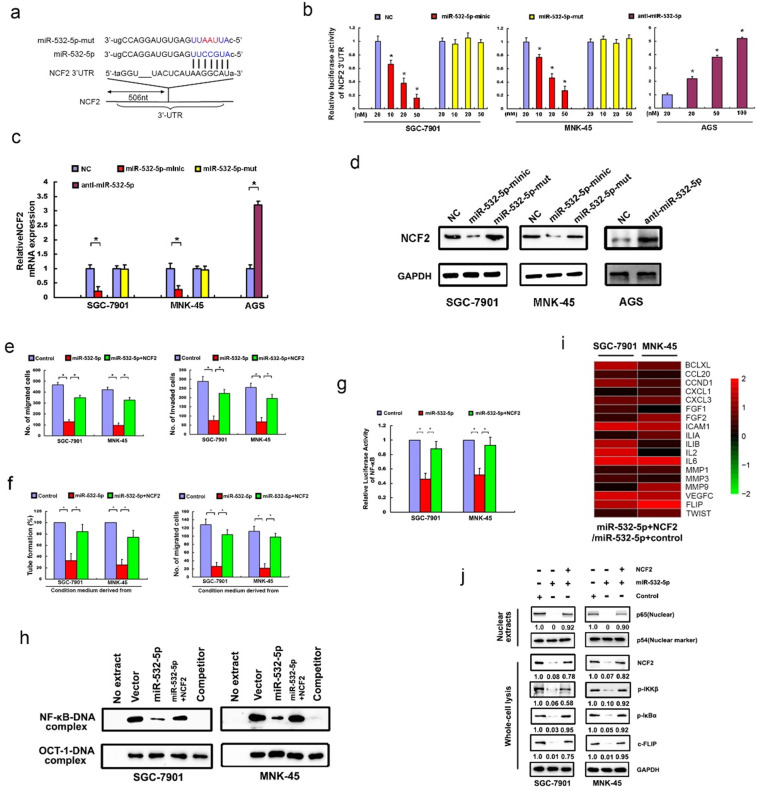
NCF2 is the direct target of miR-532-5p and affects the in vitro function of miR-532-5p in GC cells. **a** The predicted target sequence of miR-532-5p in 3′UTR of NCF2 (NCF2-3′UTR) and mutant containing three altered nucleotides in the seed sequence of miR-532-5p (miR-532-5p-mut). **b** Luciferase assay of pGL3-NCF2-3′-UTR reporters in the presence of increasing amounts (10, 20, 50 nM) of miR-532-5p mimic and mutant oligonucleotides, or increasing amounts (20, 50, 100 nM) of miR-532-5p inhibitor oligonucleotides in GC cell line. qRT-PCR analysis (**c**) and western blot analysis (**d**) showed miR-532-5p tranfection decreased NCF2 mRNA and protein level in SGC-7901 and MNK-45 cells, while anti-miR-532-5p dramatically increased NCF2 mRNA and protein level in AGS cell. **e** Transwell migration assay (Left) and Matrigel invasion assay (Right) showed the migratory capacity and invasion ability of miR-532-5poverexpressing SGC-7901 and MNK-45 cells was strengthened when transfected with full-length NCF2. **f** Restoration of NCF2 compromised the inhibitory effects of miR-532-5p on the abilities of capillary tube formation (Left) and in vitro migration (Right) of HUVECs. **g** Expression of NF-κB luciferase reporter activities were determined in the indicated cells. **h** EMSA showed that restoration of NCF2 counteracted the inhibitory effect of miR-532-5p on endogenous NF-κB activity. qRT-PCR (**i**) and Western blotting (**j**) assay indicated that overexpression of NCF2 compromised the suppression effect of miR-532-5p on numerous NF-κB-targeted genes and proteins.

**Fig. 5:**
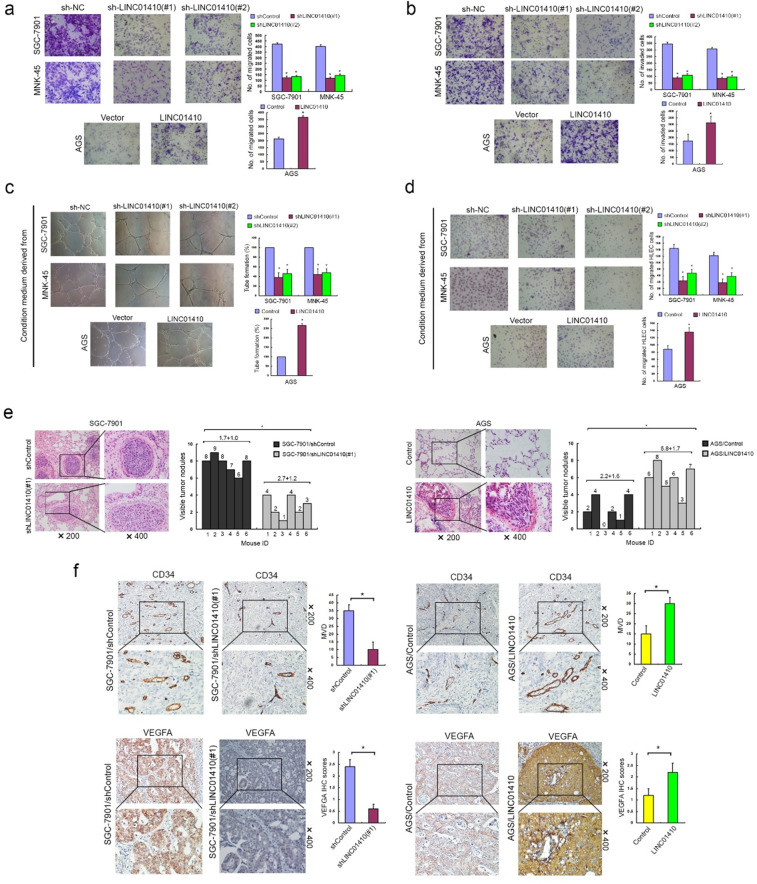
LINC01410 promotes GC metastasis and angiogenesis in vitro and in vivo. Enforced overexpression of LINC01410 largely promoted, while inhibition of LINC01410 potentially compromised the migratory (**a**) and invasion (**b**) ability of GC cells. The abilities of in vitro capillary tube formation (**c**) and migration (**d**) of HUVEC were largely compromised after incubating with culture medium of LINC01410-transfected AGS cell, while potentially strengthened after incubating with culture medium of LINC01410silenced SGC-7901 and MNK-45 cell. **e** Overexpression of LINC01410 increased, while silencing LINC01410 inhibited in vivo metastatic ability of GC cell. **f** The VEGFA levels and MVD in tumor tissue of nude mice models with subcutaneous implantation of GC were significantly decreased when LINC01410 was downregulated in SGC-7901, while largely increased when LINC01410 was upregulated in AGS cells.

